# Field-Assisted Sintering/Spark Plasma Sintering of Gadolinium-Doped Ceria with Controlled Re-Oxidation for Crack Prevention

**DOI:** 10.3390/ma13143184

**Published:** 2020-07-16

**Authors:** Tarini Prasad Mishra, Alexander M. Laptev, Mirko Ziegner, Sree Koundinya Sistla, Anke Kaletsch, Christoph Broeckmann, Olivier Guillon, Martin Bram

**Affiliations:** 1 Institute of Energy and Climate Research: Materials Synthesis and Processing (IEK-1), Forschungszentrum Jülich, 52425 Jülich, Germany; t.mishra@fz-juelich.de (T.P.M.); o.guillon@fz-juelich.de (O.G.); m.bram@fz-juelich.de (M.B.); 2 Institute of Energy and Climate Research: Microstructure and Properties of Materials (IEK-2), Forschungszentrum Jülich, 52425 Jülich, Germany; m.ziegner@fz-juelich.de; 3 Institute for Materials Applications in Mechanical Engineering (IWM), RWTH Aachen University, 52062 Aachen, Germany; s.sistla@iwm.rwth-aachen.de (S.K.S.); a.kaletsch@iwm.rwth-aachen.de (A.K.); c.broeckmann@iwm.rwth-aachen.de (C.B.); 4 Jülich Aachen Research Alliance, JARA-Energy Section, 52425 Jülich, Germany

**Keywords:** gadolinium-doped ceria, spark plasma sintering, chemical expansion, re-oxidation, crack-free sintering

## Abstract

Gadolinium-Doped Ceria (GDC) is a prospective material for application in electrochemical devices. Free sintering in air of GDC powder usually requires temperatures in the range of 1400 to 1600 °C and dwell time of several hours. Recently, it was demonstrated that sintering temperature can be significantly decreased, when sintering was performed in reducing atmosphere. Following re-oxidation at elevated temperatures was found to be a helpful measure to avoid sample failure. Sintering temperature and dwell time can be also decreased by use of Spark Plasma Sintering, also known as Field-Assisted Sintering Technique (FAST/SPS). In the present work, we combined for the first time the advantages of FAST/SPS technology and re-oxidation for sintering of GDC parts. However, GDC samples sintered by FAST/SPS were highly sensitive to fragmentation. Therefore, we investigated the factors responsible for this effect. Based on understanding of these factors, a special tool was designed enabling pressureless FAST/SPS sintering in controlled atmosphere. For proof of concept, a commercial GDC powder was sintered in this tool in reducing atmosphere (Ar-2.9%H_2_), followed by re-oxidation. The fragmentation of GDC samples was avoided and the number of micro-cracks was reduced to a minimum. Prospects of GDC sintering by FAST/SPS were discussed.

## 1. Introduction

Gadolinium-doped ceria (GDC) is a well-established material for many chemical and electrochemical processes. Due to its specific properties, GDC is widely applied as catalyst of chemical reactions [1]. Furthermore, higher ionic conductivity at temperatures in the range 500 to 600 °C makes GDC attractive to be applied as electrolyte in Intermediate Temperature Solid Oxide Fuel Cells (IT SOFC) instead of commonly used yttria-stabilized zirconia (YSZ). Nowadays, it is in use in commercialized metal-supported IT SOFC systems of the British company Ceres Power and its shareholder Bosch [2,3]. Furthermore, it has potential as electrochemically active membrane material for oxygen transport membranes [4]. Recently, our group demonstrated that GDC increases electrochemical activity of Ni-based cermet anodes [5,6] as well as mechanical stability of perovskite cathodes in SOFC [7], making this material a bright future prospect for manifold applications in the chemistry and energy sector. However, processing of GDC as bulk or membrane material is a challenging task, which usually requires either high temperatures or careful atmosphere control as discussed in more detail below. The complexity of processing GDC becomes even higher, when it is in contact with other functional materials, which is the usual case in electrochemical devices, e.g., in cermet electrodes [5,6,7] or dual phase membranes [4]. In this work, the focus will lie on problems and solutions in processing of pure GDC by Field-Assisted Sintering Technique, also known as Spark Plasma Sintering (FAST/SPS). In a more general way, the main findings of this work can be transferred to other oxide ceramics with high catalytic activity. Examples are materials for oxygen transport membranes like lanthanum strontium cobalt oxides or materials for electrolytes in all-solid-state batteries like lithium-containing zirconates or phosphates. For this group of materials, high catalytic activity or high ionic conductivity is often coupled with a limited thermodynamic stability, which again makes processing of such materials challenging with respect to phase purity, chemical expansion and chemical reaction with other compounds in multilayered systems.

In the specific case of GDC, increased catalytic activity and enhanced ionic conductivity is achieved by doping of CeO_2_ with Gd_2_O_3_. The partial replacement of Ce^+4^ ions by Gd^+3^ ions in cubic (fluorite) lattice of CeO_2_ leads to formation of oxygen vacancies. The enhanced concentration of vacancies in GDC eases the diffusion of oxygen ions through the lattice, therefore increasing the ionic conductivity. The chemical composition of GDC can be described by formula Ce_1-*x*_Gd*_x_*O_2-*x*/2_. Here, *x* is the molar amount of Gd_2_O_3_ dopant. The maximum concentration of Gd_2_O_3_ usually does not exceed 20%. A further dopant increase is not beneficial for both ionic conductivity and mechanical stability of GDC [8]. In practical applications, mainly two compositions with *x* = 0.1 (GDC10) and *x* = 0.2 (GDC20) are used.

Despite certain differences, GDC and other rare-earth doped cerium oxides inherit from pristine CeO_2_ several interrelated features which are important for understanding sintering by FAST/SPS technique. Firstly, GDC can be easily transferred to a non-stoichiometric state due to loss of oxygen atoms in a reducing atmosphere or in vacuum. Apparently, the reaction of GDC10 with hydrogen can be described as

Ce_0.9_Gd_0.1_O_1.95_+ δH_2_ = Ce_0.9_Gd_0.1_O_1.95-δ_ + δH_2_O (1)

The proposed reaction (1) is similar to the reaction between undoped ceria and hydrogen presented in the literature [9,10]. Here, a part of Ce^+4^ ions in the lattice converts to Ce^+3^ generating oxygen vacancies and free electrons. In this equation, δ represents the molar amount of oxygen per mole of GDC10. This value will be denoted further as index of non-stoichiometry. The related generation of oxygen vacancies and electrons can be described as

δO^2−^ = δV_o_ + 2δ*e*^−1^ + δ/2O_2_ (2)

The symbols V_o_, *e*^−1^ and O^2−^ designate oxygen vacancy, electron and oxygen ion, respectively. In an oxygen rich environment, GDC10 spontaneously picks up oxygen according to reaction (3) towards the initial stoichiometric composition.

2Ce_0.9_Gd_0.1_O_1.95−δ_ + δO_2_ = 2Ce_0.9_Gd_0.1_O_1.95_ (3)

Analogous redox transformation occurs in other reducing atmospheres. For instance, GDC10 can interact with carbon monoxide (CO) according to reaction (4). We have formulated this reaction similar to the well-known reaction of non-doped ceria and carbon monoxide [10].

Ce_0.9_Gd_0.1_O_1.95_ + δCO = Ce_0.9_Gd_0.1_O_1.95−δ_ + δCO_2_ (4)

The generation of oxygen vacancies and free electrons during reduction increases both ionic and electronic conductivity of GDC. Theoretically, the value of non-stoichiometry index δ can vary from 0 to 0.5. However, in reality the maximal value of δ depends on oxygen partial pressure and temperature. Usually, non-stoichiometry index does not exceed a value of 0.3. At higher degree of non-stoichiometry, GDC decomposes into different secondary phases with a drastic decrease in ionic conductivity [11]. Reduction of GDC by hydrogen and carbon monoxide and subsequent re-oxidation is well studied at temperatures up to 600 °C, which are representative for IT SOFC operation [12]. In the present paper we investigated this process at temperatures up to 1050 °C, which are more relevant to sintering conditions.

Another important peculiarity of GDC at low oxygen partial pressures is chemical expansion in the case of partial reduction and related change in stoichiometry. Referring to the fundamental work of Hong et al. [13], the physical reason behind chemical expansion is superposition of two effects coupled with the formation of defects during reduction: (a) the increase of ionic radius upon decrease in valence state of the cations from Ce^4+^ (0.907 Å) to Ce^3+^ (1.143 Å) and (b) the formation of positively charged oxygen vacancies with subsequent electrostatic repulsion of the surrounding cations. Brauer and Gingerich found for undoped CeO_2-δ_ nearly linear dependence of chemical expansion on non-stoichiometry index *δ* [14]. Bishop et al. reported for GDC10 a chemical expansion of about 10∙δ (in %) at 800 °C [15]. Inhomogeneous chemical expansion in a dense GDC electrolyte can lead to large mechanical stresses, crack formation and disintegration of the material [16]. The inherent risk of GDC to reduction with related chemical expansion is one of the reasons for limited application of GDC as electrolyte in IT SOFCs, where hydrogen or other reducing gases come in contact with the electrolyte [12]. It should be noted that chemical expansion is a reversible process and can be revoked by controlled re-oxidation treatment.

In addition, proceeding reduction of GDC leads to change of colour from bright to dark appearance. This feature is also known for other oxide ceramics (e.g., ZrO_2_) as “blackening” [17]. The shading and intensity of color change depends on the non-stoichiometry of GDC. The blackening effect was investigated in detail for CeO_2_ by Bevan [18]. In his paper, the color change from initial pale yellow to gray blue, dark blue, blue-black and black was directly related to the non-stoichiometry. A similar color change is observed during reduction of GDC. Thus, the color of GDC can serve to some extent as an indicator of its reduction state.

In most cases, bulk GDC is manufactured by powder compaction with subsequent free sintering in air. The sintering is usually performed at a temperature in the range of 1400 to 1600 °C and with a dwell time varying from 30 min to several hours. Optimum processing conditions depend on properties of starting powder like primary particle size. For instance, Neuhaus et al. reported a temperature of 1600 °C and a holding of 8 h necessary for sintering of commercial GDC10 (Sigma-Aldrich, St. Louis, MO, USA) powder to a relative density of 96.3% [19]. The application of a reducing sintering atmosphere instead of air significantly enhances the densification rate and grain growth in GDC. This effect was investigated mainly for H_2_-H_2_O and H_2_-N_2_ atmospheres [11,20,21,22]. In our paper the H_2_-Ar atmosphere was additionally proposed. The mechanism of sintering kinetics in reducing atmosphere was studied in detail by Esposito et al. [23]. The authors concluded that the main reason for increase of sintering activity in reducing atmosphere is the formation of oxygen vacancies due to the stoichiometry change as already introduced by Equations (1) and (2). According to Esposito et al., the related reaction predominantly occurred on grain boundaries. This result is in line with traditional understanding of sintering in oxide ceramics as detailed discussed in recognized text books [24]. At first glance it may appear that reducing atmosphere can be used for decrease of sintering temperature. However, a close look at the densification curves in air and in hydrogen-containing atmosphere shows that this is not fully true. Indeed, the onset temperature of densification is lower and densification rate in reducing atmosphere is, in general, higher. However, the latter is correct only at initial and intermediate stage of densification. At a higher density—exceeding a value of around 95% of theoretical value—the advantage of reducing atmosphere can vanish. Here, the sintering in air at high temperature (e.g., 1450 °C) becomes faster due to a specific de-densification effect in reducing atmosphere [23]. This effect can be attributed to accumulation of vacancies with formation of intergranular micro-pores due to continued transformation of Ce^+4^ to Ce^+3^. However, at such densities GDC is already rather gas tight, as it is required in most applications. A decrease in sintering temperature can reduce or probably fully exclude de-densification. In particular, Esposito et al. reported free sintering of a fine GDC10 powder to almost theoretical density in 9% H_2_-N_2_ atmosphere at a temperature of 1050 °C and with a dwell time of 60 min [23]. However, after sintering in a reducing atmosphere formation of micro-cracks and fragmentation of samples was observed. The authors attributed this phenomenon to interaction of non-stoichiometric GDC with oxygen from air during or after cooling. A result of such an interaction is the rapid re-oxidation of GDC with related non-homogeneous chemical contraction. In worst case, this effect can lead to large stress level and, in combination with weakened grain boundaries, to mechanical disintegration of sintered material. To avoid such a scenario, Ni at al. proposed the controlled re-oxidation of GDC during cooling [21]. The authors used re-oxidation firstly in nitrogen and then in oxygen at a temperature in the range of 800 to 1200 °C. With this measure, maintaining mechanical integrity of GDC after free sintering was achieved. However, this concept was not proven for sintering techniques other than free sintering, for instance for FAST/SPS.

Besides, pressure-assisted sintering is another way to decrease the sintering temperature of ceramics including GDC. In particular, Spark Plasma Sintering, also referred to in the literature as Field Assisted Sintering Technique (FAST/SPS), can be used for this aim [25]. The basic principles and applications of this technology were described in numerous reviews and research papers [26,27,28,29]. Typical characteristics of conventional FAST/SPS technique are: (a) fast resistive heating by pulsed direct current; (b) use of graphite tools combined with other graphite-based components such as foil, felt etc.; (c) application of an axial pressure of 30–100 MPa depending on used graphite quality; (d) sintering in a moderate vacuum of around 1 mbar or under protective atmosphere of nitrogen, argon or helium; (e) water cooling of sintering chamber and electrodes; (f) well-controlled temperature and pressure profile including cooling stage. All these parameters are just guide values and can be modified with respect to the specific application, e.g., by using alternative tool materials or specifically equipped FAST/SPS devices. For instance, replacing graphite tool by a tool manufactured from titanium-zirconium-molybdenum (TZM) alloy enables significant increase of applied pressure [30]. In particular, Groeneveld et al. reported sintering of Cr_2_S_3_ powder in a TZM tool at a pressure of 395 MPa and at a temperature of 950 °C [31]. However, the use of such a tool is apparently limited by a maximum temperature of around 1200 °C, at which recrystallization of TZM starts causing the abrupt decrease of mechanical strength [32].

Spark plasma sintering of GDC is described only in a few papers. More papers are dedicated to FAST/SPS of undoped ceria or ceria doped with other rare-earth elements. Moreover, information on the ability of crack-free processing of GDC and similar oxides by FAST/SPS is controversial. In general, published results can be divided into two groups. One group of authors did not mention any problem with respect to the integrity of GDC samples after FAST/SPS processing. The other group reported on difficulties of avoiding crack formation and on severe fragmentation of FAST/SPS sintered GDC samples. However, reliable achieving full integrity of sintered material is a crucial point for any application. To clarify the state-of-the-art, main findings of both groups are summarized in compact form below.

The first group of papers deals with the sintering of both pure and doped ceria. Anselmi-Tamburini et al. reported successful FAST/SPS sintering of CeO_2_ and Ce_0.7_Sm_0.3_O_1.85_ starting from nanometer sized powders (primary particle size <10 nm). Sintering was done at temperatures of 625 °C and 750 °C applying pressures of 600–610 MPa. In both cases, dwell time at sintering temperature was 5 min [33]. Exceptionally large pressures were achieved by using a special FAST/SPS tool with SiC punches and WC protection inserts. The samples were nearly dense (>98%), but small in size with a diameter of 5 mm and a thickness of 1–3 mm. Choi et al. used the same tool to investigate the influence of dopant content on FAST/SPS sintering of Dy_2_O_3_-doped CeO_2_ nanometric powder (primary particle size <73 nm) [34]. The dopant amount was varied between 3 and 10 wt.%. FAST/SPS was done at a pressure of 500 MPa and a temperature of 900 °C and 1050 °C. A maximal density of 97% was achieved with 10 wt.% of Dy_2_O_3_ after sintering at 1050 °C with a dwell time of 5 min. Mori et al. sintered Ce_0.8_Dy_0.2_O_1.9_ powder with an average primary particle size of 20 nm in a graphite die (15 mm in diameter) at temperatures in the range of 1000 °C to 1200 °C applying a pressure of 60 MPa [35]. The maximal attained density was around 85%. The authors attributed this low density value to penetration of carbon from the graphite die into the specimens. Therefore, the holding at sintering temperature was omitted. As secondary operation, conventional sintering in air was done to reach the desired density above 95%. Solodkyj et al. sintered an in-house synthesized Ce_0.8_Sm_0.2_O_1.9_ powder with an average agglomerate size of 47 nm and a primary crystallite size of 7 to 11 nm in conventional graphite FAST/SPS tool with a die of 10 mm in diameter [36]. A temperature of 1000 °C, pressure of 150 MPa and holding time of 10 min were applied resulting in a relative density of 95%. Argon atmosphere was used apparently to suppress the powder decomposition. However, the electrochemical performance was significantly lower compared to conventional sintered samples. Vasylkiv et al. used similar approach for sintering of a Ce_0.8_Gd_0.2_O_1.9_ powder in a graphite die with a diameter of 10 mm [37]. A primary particle size of the in-house synthesized powder was around of 5–7 nm. A relative density of above 98% was achieved after sintering at 1050 °C, with a dwell time of 5 min and a pressure of 150 MPa. Sintering was performed in a moderate vacuum of around 0.45 bar. Shimonosono et al. applied an even lower temperature of 900 °C for sintering of Ce_0.8_Gd_0.2_O_1.9_ at a pressure of 90 MPa and with 5 min holding [25]. Despite low sintering temperature and relatively small pressure, a density of 96.2% was achieved. The starting powder with particle size less than 20 nm was synthesized by authors. Recently, Kabir et al. sintered an in-house synthesized Ce_0.9_Gd_0.1_O_1.95_ powder with a primary particle size of 12 nm by FAST/SPS. In this case, a final relative density of around 96% was achieved [38]. The sintering was performed at 980 °C, under a pressure of 70 MPa and with a dwell time of 5 min. In this work, the authors did not report any crack formation or fragmentation of samples. Table 1 summarizes the FAST/SPS parameters, primary particle sizes and resulting sintering densities reported in the first group of papers.

Contrary to the first group, severe crack formation and fragmentation of samples into several pieces were reported in the following works. Watkinson et al. investigated conventional and FAST/SPS sintering of CeO_2_ as analogue for AmO_2_ [39]. Commercial CeO_2_ powder (Sigma-Aldrich, St. Louis, MO, USA) with a micrometer particle size <5 µm was used as starting material. Samples with a diameter of 20 mm and height in the range of 3.5–4.5 mm were sintered by FAST/SPS in vacuum at temperatures in the range of 1100–1500 °C. Density above 90% was only achieved after sintering at 1500 °C, applying a pressure of 80 MPa and a dwell time of 3 min. However, the sintered pellets were disintegrated into fragments. The authors even concluded that FAST/SPS is not a suitable technology to sinter CeO_2_. Prasad et al. investigated FAST/SPS of other commercial CeO_2_ powder (ACROS Organics) with a particle size of ~20 µm [40,41]. After sintering at 1400 °C, with 60 MPa pressure and 5 min dwell, a relative density of 91% was achieved. The pellets were 20 mm in diameter. Though the pellets were visually stable, they can be easily fractured manually. This behavior indicated the presence of numerous micro-cracks. The data reported in the second group of papers were summarized in Table 2.

Comparison between FAST/SPS parameters in Table 1 and Table 2 leads to the conclusion that the sintering temperature is a decisive parameter for crack-free sintering of GDC and similar ceramics. Apparently, sintering temperature should be reduced to 1050 °C or below. This can be preferentially achieved by the use of powders with higher sintering activity due to small primary particle sizes in nanometer range. The sintering temperature can be further reduced by application of exceptionally high pressure in the range of 500 to 600 MPa, which is about 10 times larger than usually applied during FAST/SPS. It becomes obvious that lowering of FAST/SPS temperature is an appropriate option to keep the reduction of GDC during FAST/SPS processing acceptably low. However, the lessened reduction of GDC can be achieved by other measures as well, which has not been reported in the literature so far. In particular, physical contact between GDC powder and graphite components of FAST/SPS setup could be reduced or avoided by suitable tool design. As an alternative, a graphite-free tool can be discussed. Then, probably, a coarser, i.e., micrometer sized GDC powder could be successfully processed by FAST/SPS as well. Such powders are usually cheaper, commercially available and easier in handling as compared with nanometric powders. Furthermore, commercial and quality assured powders would be the first choice at applying FAST/SPS to manufacturing of electrochemical devices at industrial scale.

The aim of the present work is to draw sound conclusions on the potential of FAST/SPS technology for crack-free sintering of materials, which are prone to reaction with FAST/SPS graphite tool, release of oxygen and chemical expansion. For the study, we chose GDC as the example due to representing this group of materials in a reasonable way while being already applied in commercial electrochemical devices. With this work, we are aiming on going a step beyond the partly controversial and incomplete discussion in the literature on challenges related to handling this group of materials. Here, our focus lies on FAST/SPS processing of commercial, micrometer sized GDC powder. Firstly, all factors being responsible for cracking of GDC pellets at conditions typical for FAST/SPS sintering are investigated by related experiments. In this context, the chemistry and physics of underlying processes will be highlighted and discussed in detail. Based on our findings, a new concept of FAST/SPS tool is proposed, which enables sintering of even thin, free standing membranes. Specific design of this tool excluding direct contact of GDC with graphite and enabling for the first time control of atmosphere in vicinity of sintered sample in a FAST/SPS device was suggested. The suitability of new tool design for crack-free FAST/SPS of GDC powder was demonstrated. In our future work, the results will act as basis for establishing FAST/SPS sintering as promising supplemental method for synthesis of GDC-based components and composites for electrochemical devices, e.g., electrolytes and electrodes for solid oxide cells and dual phase oxygen transport membranes. Lowering sintering temperature and dwell time by applying FAST/SPS in combination with controlled atmosphere is supposed to be especially attractive for co-firing of GDC in contact with other electrochemically active materials.

## 2. Experimental

### 2.1. Initial Powder

**Figure 1:**
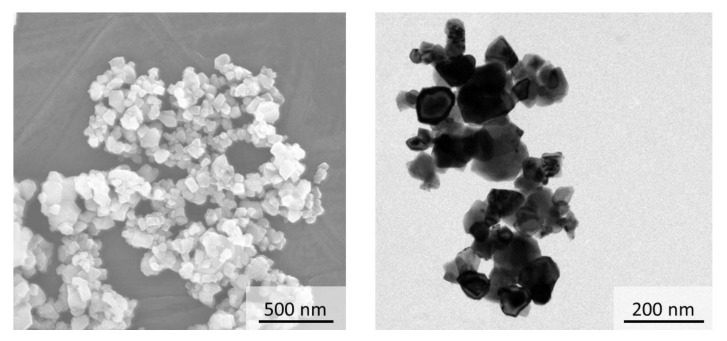
Morphology of GDC10-HP powder: (**a**) Scanning Electron Microscopic (SEM) image; (**b**) Transmission Electron Microscopic (TEM) image.

Commercial Ce_0.9_Gd_0.1_O_1.95_ powder, grade GDC10-HP (FCM, Columbus, OH, USA) was used for all experiments. The supplier reported for the batch used in this study a normal particle size distribution with d_10_ = 0.12 µm, d_50_ = 0.21 µm, and d_90_ = 0.46 µm. In-house measurement of particle size by Horiba LA-950 (Retsch, Haan, Germany) confirmed these values. However, the powder tended to form agglomerates with a size in the range of 1 to 10 µm. A specific surface area of 10.5 m^2^·g^−1^ was determined in-house by Area Meter II device (Ströhlein Instruments, Kaarst, Germany). Again, this value is in line with the data provided by the supplier. Scanning Electron Microscopic (SEM) (Ultra 55, Carl Zeiss, Jena, Germany) and Transmission Electron Microscopic (TEM) (FEI Titan G2 80-200, FEI, Hillsboro, OR, USA) images of the as-received powder are shown in Figure 1. The color of starting powder was pale yellow. In summary, the particle size of the used powder lay between values typical for nanometer and micrometer sized powders (see also Table 1 and Table 2).

### 2.2. Dilatometry

The influence of atmosphere on densification behavior of GDC10-HP powder was examined in a push rod dilatometer TMA 402 F1 (Netzsch, Selb, Germany). The experiments were performed in air, argon and Ar-2.9% H_2_ atmosphere with a constant gas flow of 50 mL∙min^−1^. The dilatometer samples were manufactured by uniaxial compaction with a pressure of 110 MPa. The cylinder-shaped samples had a diameter of 8 mm, height of 4 to 5 mm and relative density of around 55%. The dilatometer cycle included a heating ramp with 3 °C∙min^−1^ to 1400 °C, a dwell time at 1400 °C for 30 min and then cooling to room temperature with 3 °C∙min^−1^. During sintering of GDC samples, measured displacement included densification due to sintering, thermal expansion and chemical expansion. To exclude the influence of thermal expansion, its contribution was subtracted from measured shrinkage. In doing so, a thermal expansion coefficient (TEC) of 14.7 × 10^−6^ °C^−1^ for sintering in air and 13.4 × 10^−6^ °C^−1^ for sintering in Ar-2.9% H_2_ was used. These TEC values were determined from shrinkage of dense samples during cooling in dilatometer. Hence, the shrinkage curves in this paper represent the algebraic sum of shrinkage caused by sintering and chemical expansion due reduction of material, which is significantly pronounced in Ar-2.9% H_2_. Additionally, dilatometer experiment with change of atmosphere from Ar-2.9% H_2_ to air during cooling was performed. Here, the sample was firstly heated up to a temperature of 1400 °C in Ar-2.9% H_2_ atmosphere. After holding for 30 min the sample was cooled down to a temperature of 800 °C. At this temperature, the atmosphere was switched to air. The sample was held for 10 min at this temperature and then cooled to room temperature. With this experiment, the influence of re-oxidation at elevated temperature on structural integrity of sintered GDC10-HP samples was verified.

### 2.3. Thermal Gravimetric and Spectral Analysis

Thermal gravimetric analysis was used for studying the re-oxidation behavior of GDC10-HP powder and for examination of its reaction with graphite. The experiments were performed in a STA 449 F1 device (Netzsch, Selb, Germany) coupled with mass spectrometer QMS 403 Aëolos (Netzsch, Selb, Germany). During re-oxidation experiments, a small amount of GDC10-HP powder was firstly heated up with 3 °C∙min^−1^ to 1050 °C in Ar-2.9% H_2_ flow. Then, the sample was held at this temperature for 30 min and cooled down with 3 °C∙min^−1^ to room temperature. At this point, Ar-2.9% H_2_ atmosphere was changed to air and the sample was held at room temperature for 30 min. Subsequently, the sample was heated again with the same rate to 1050 °C in air flow. After holding at this temperature for 30 min, the sample was cooled down with 3 °C∙min^−1^ to room temperature. The mass change was monitored during entire cycle. For studying reduction of GDC10-HP power in the case of being in contact with graphite, GDC powder was mixed with nanometer sized carbon black powder. The small amount of this mixture was placed in an alumina crucible and heated up with 3 °C∙min^−1^ to 1400 °C in argon. After holding at this temperature for 30 min, the mixture was cooled down to room temperature with 3 °C∙min^−1^. In this experiment, mass change was monitored and gas composition was analyzed by spectrometer.

### 2.4. High-Temperature X-ray Diffraction Analysis

Chemical expansion in reducing Ar-2.9%H_2_ atmosphere was studied by high-temperature X-ray diffraction (HT-XRD) technique. In this experiment, the diffraction patterns were recorded at different temperatures during heating the as-received powder. The investigation was performed in Empyrean diffractometer (Malvern Panalytical, Malvern, UK) in the scanning range of 2θ between 25° and 145° using CuKα radiation. The sample was heated with 3 °C∙min^−1^ to 1000 °C. XRD measurements were performed at 25 °C, 100 °C, 200 °C, 300 °C, 400 °C and from 400 °C to 1000 °C at each 50 °C interval. At temperatures above 200 °C, a dwell time of 20 min was applied during each measurement to ensure thermal stabilization. All measurements were repeated in synthetic air (Ar-20%O_2_) for determining thermal expansion of the lattice. The lattice parameter was calculated based on diffraction peaks position with Bragg’s law. Rietveld refinement of XRD pattern and TOPAS software (Bruker AXS, Karlsruhe, Germany) were used. The crystal structure of Gd_0.10_ Ce_0.90_ O_1.95_ was taken from the Inorganic Crystal Structure Database (ICSD collection code 28795, FIZ Karlsruhe, Karlsruhe, Germany). The peaks width tended to decrease with increasing temperature due to growing crystallite size. The estimated error for lattice parameter was about 0.001 Å. The chemical expansion was calculated by extracting thermal expansion of the lattice measured in Ar-20%O_2_ from the lattice parameters measured in Ar-2.9%H_2_.

### 2.5. Spark Plasma Sintering Technique

Spark plasma sintering was performed in a HP D5 device (FCT Systeme, Rauenstein, Germany) in vacuum, entirely in Ar-2.9%H_2_ flow or in Ar-2.9%H_2_ flow with subsequent change to Ar-20%O_2_ (synthetic air) during cooling (redox FAST/SPS). To accomplish this change, the gas system was slightly modified. It is worthy to note that Ar-2.9%H_2_ and Ar-20%O_2_ are not the standard gases in FAST/SPS devices. In our experiments Ar-2.9%H_2_ was taken from the central gas distribution system of the institute. The synthetic air was supplied from an external gas bottle. A special switcher enabled manual change between both atmospheres. The oxygen partial pressure was measured by a SGM7 oxygen analyzer (ZIROX Sensoren & Elektronik GmbH, Greifswald, Germany) positioned at the gas outlet.

**Figure 2:**
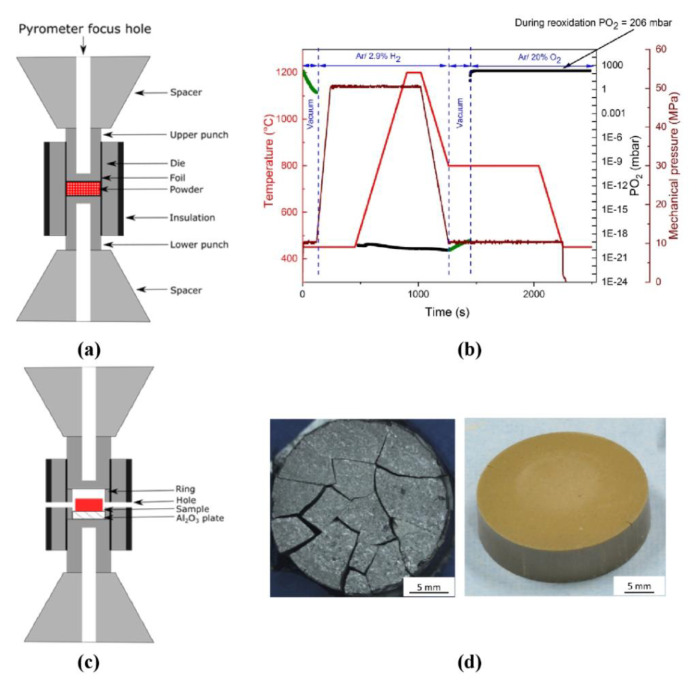
(**a**) Setup for pressure-assisted FAST/SPS; (**b**) redox cycle; (**c**) setup for pressureless FAST/SPS; (**d**) fractured sample after pressure-assisted sintering (left) and sample after pressureless redox sintering (right).

Two different sintering setups were used. The first setup provided regular pressure-assisted FAST/SPS sintering in a graphite tool. This setup is schematically shown in Figure 2a. The setup included the die with a diameter of 20 mm, two punches and two conical spacers. All components were manufactured from graphite R7710 (SGL Carbon, Bonn, Germany). A graphite foil with a thickness of 0.34 mm (SGL Carbon, Bonn, Germany) was placed between powder and tool components to ensure better electrical and thermal contact and to avoid sticking when removing the sample. The die was thermally insulated by graphite felt (SGL Carbon, Bonn, Germany) with a thickness of 11.5 mm to homogenize the temperature and to reduce the energy consumption by diminishing heat radiation [42]. The temperature was controlled by an axial pyrometer (Pyrospot DG 10N, DIAS Infrared GmbH, Dresden, Germany) focused on the bottom of blind drilling in the upper punch.

Portions of 6 g of GDC10-HP powder were poured into the die and pre-compacted with a pressure of 50 MPa. Then, the samples were heated up with 100 °C∙min^−1^ in vacuum, argon or in Ar-2.9%H_2_ to a sintering temperature of 1200 °C, held for 2 min and cooled with 100 °C∙min^−1^ down to 450 °C. At lower temperatures, the heating was turned off and free cooling was applied. In the case of re-oxidation experiments, the samples were initially cooled to 800 °C and then held for 13 min at this temperature (Figure 2b). During initial 3 min of this dwell, the FAST/SPS chamber was evacuated to lowest possible pressure of 0.6 mbar. Then the atmosphere was switched from Ar-2.9%H_2_ to synthetic air for rest of the cycle. A pressure of 50 MPa was applied from the beginning of the FAST/SPS cycle and gradually reduced from 50 MPa to 9.5 MPa during cooling from 1200 °C to 800 °C. The pressure was removed completely as the heating was turned off (Figure 2b).

The second setup was designed to eliminate contact between graphite components and powder compact, to avoid its clamping between punches and to improve the atmospheric control in the die cavity. This setup is schematically presented in Figure 2c. A graphite ring with an internal diameter of 24 mm was inserted in a standard die with a diameter of 30 mm. The ring formed a cavity between the punches. A cold-pressed GDC10-HP sample with a diameter of 20 mm, a height of 3.6 mm and a relative density of around 55% was placed into the cavity. To avoid interaction with graphite, the sample was placed on an alumina plate. Two 4 mm holes drilled through the die and the ring enabled gas exchange between the die cavity and the atmosphere in the FAST/SPS chamber. With this configuration, no load was applied on the sample and direct contact to graphite was reliably avoided. The dwell time during this pressureless sintering was increased to 15 min and the sintering temperature to 1400 °C to achieve density similar to conventional FAST/SPS done in first setup. All other parameters were kept constant.

### 2.6. Microstructural and Phase Analysis

The density of samples was measured by the Archimedes method. Microstructure was studied with thermally etched sample in a Phenom scanning electron microscope (FEI, Hillsboro, OR, USA). Phase analysis was performed in a D4 Endeavour XRD device (Bruker, Billerica, MA, USA).

## 3. Results

### 3.1. Influence of Atmosphere

**Figure 3:**
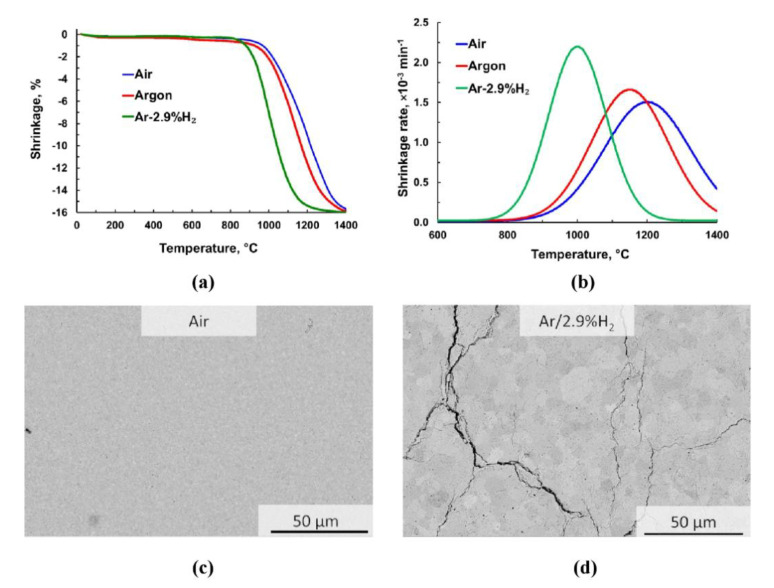
(**a**) Shrinkage of GDC10-HP samples during free sintering in different atmospheres; (**b**) associated shrinkage rate; microstructure of GDC10-HP sample sintered (**c**) in air and (**d**) in Ar-2.9%H_2_.

Relative shrinkage of GDC10-HP samples during heating in dilatometer is shown in Figure 3a. The significant influence of atmosphere on sintering kinetics becomes obvious. Lowest onset temperature of shrinkage (790 °C) and most pronounced shrinkage were observed in the case of sintering in Ar-2.9%H_2_. When sintering was done in argon or air, onset temperature of 850 °C was nearly the same for both atmospheres. Sintering kinetics were slightly faster in argon than in air, but clearly reduced compared to Ar-2.9H_2_. In Figure 3b, all shrinkage rates as function of temperature are shown. The maximum shrinkage rate in air, argon and in Ar-2.9%H_2_ was achieved at 1200 °C, 1160 °C and 1000 °C, respectively. Enhanced sinterability in Ar-2.9%H_2_ is confirmed by the lowering the temperature of maximum shrinkage rate by around 200 °C compared to sintering in air. Finally, in the case of Ar-2.9%H_2_ the densification was already completed at the end of the heating ramp. On the contrary, increased sintering temperature or longer dwell were needed to achieve the same density, when sintering in argon or air. The grain size of the sample sintered in Ar-2.9%H_2_ was approximately 2.0 ± 1.4 µm. The sample sintered in air had a grain size of 0.45 ± 0.2 µm. Thus, the use of reducing atmosphere not only shows enhanced dynamic of densification but also enhanced grain growth (approximately 5 times higher). Generation of the large oxygen vacancies concentration in the lattice during sintering under reducing atmosphere resulted in the enhanced densification and grain growth in the GDC sample. This result is in good agreement with previous findings [23]. Besides, careful analysis of the grains revealed that most of the residual pores are entrapped inside the large grains in the sample that was sintered under reducing atmosphere. Whereas, the sample which was sintered in air had the pores near to the triple points of grain boundaries.

Crack-free sintering was achieved in air (Figure 3c). In Ar-2.9%H_2_ atmosphere, drastically enhanced sintering kinetics was accompanied by crack formation (Figure 3d). It is assumed that the cracks mainly resulted from chemical expansion due to reduction and subsequent rapid re-oxidation of Ce_0.9_Gd_0.1_O_1.95_ when opening the dilatometer chamber. Uncontrolled contact to air at ambient conditions is expected to cause cracking due to spontaneous and non-homogeneous contraction of the sample. The appearance of cracks after sintering in Ar-2.9%H_2_ atmosphere was successfully suppressed by re-oxidation at 800 °C with feeding synthetic air during the subsequent cooling stage and keeping this temperature for 10 min. This result is in good agreement with the literature, where authors performed free sintering of Ce_0.9_Gd_0.1_O_1.95_ in different atmospheres (including re-oxidation) starting from nanometer sized powders [20,21,22,23]. Thus, the positive effect of re-oxidation on integrity of free sintered GDC10-HP samples was further evidenced.

### 3.2. Reduction and Re-oxidation

**Figure 4:**
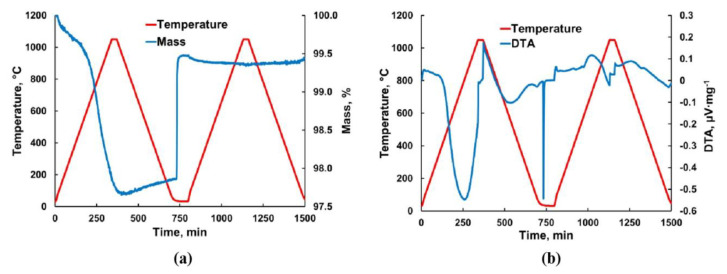
(**a**) Mass change and (**b**) differential thermal analysis of GDC10-HP powder during reduction in Ar-2.9%H_2_ in first thermal cycle and re-oxidation in air in second thermal cycle.

The ability of GDC10-HP to release and to adopt oxygen was studied by thermal gravimetric analysis. The mass change during two consecutive thermal cycles is shown in Figure 4. The initial mass loss of around 0.41% during heating in Ar-2.9%H_2_ to 500 °C can be associated with the evaporation of moisture. The following decrease in the mass was caused by reduction of Ce_0.9_Gd_0.1_O_1.95_ in contact with hydrogen according to reaction (1). When heating to 1050 °C in the first cycle, a total mass loss of 2.3% was measured. Thus, a mass loss of approximately 1.89% can be related to release of oxygen clearly changing the non-stoichiometry index δ. The δ value can be calculated by Equation (5):

(5) $$\delta =\Delta m\;\cdot \;\frac{M_{GDC10}}{M_{O}}$$

Here, Δ*m* is the relative mass loss, *M_GDC_*_10_ is the molecular mass of Ce_0.9_Gd_0.1_O_1.95_ and *M_O_* is the atomic mass of oxygen. In our case, the non-stoichiometry index after heating to 1050 °C was calculated to 0.204. During subsequent cooling in the first cycle in Ar-2.9%H_2_, a small mass gain of around 0.19% was observed (Figure 4a). This can be caused by partial incorporation of oxygen back into the Ce_0.9_Gd_0.1_O_1.95-δ_ lattice according to reaction (3) when lowering the temperature. It is worth to note that this process occurred at very low oxygen partial pressure hinting on strongly pronounced activity of non-stoichiometric Ce_0.9_Gd_0.1_O_1.95-δ_ for picking up oxygen from surrounding atmosphere. Therefore, sudden re-oxidation of Ce_0.9_Gd_0.1_O_1.95-δ_ when feeding air into the sintering chamber at room temperature is not surprising. Rapid oxygen uptake was indicated by a mass gain of 1.57% and a sharp exothermal peak in differential thermal analysis (DTA) diagram (Figure 4b). The latter means that a reduced GDC sample can rapidly heat up to elevated temperature when suddenly exposed to oxygen. In practice, we observed such a self-sustaining heating of the sample after ejecting from the FAST/SPS tool under ambient conditions. This finding is important for understanding the reasons of crack formation when processing GDC via FAST/SPS technique.

### 3.3. Chemical Expansion

**Figure 5:**
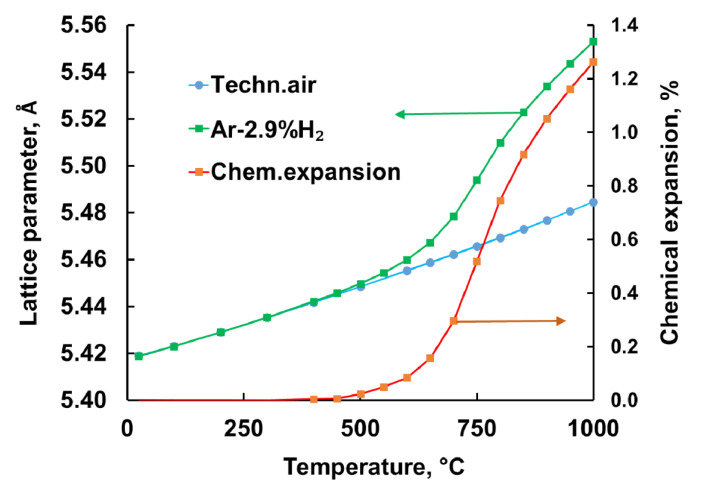
Expansion of GDC10 lattice during heating in synthetic air and in Ar-2.9%H_2_.

The results of HT-XRD analysis are shown in Figure 5. Relationship between lattice parameter and temperature during heating and cooling in synthetic air remained almost the same. Therefore, in this case, change of lattice parameters can be solely attributed to thermal expansion. In the investigated temperature range, the relationship between thermal expansion and temperature was nearly linear. This behavior changed when GDC10-HP was heated in Ar-2.9%H_2_. The deviation from linearity started at around 500 °C. In the following, this temperature is defined as onset temperature for reduction of GDC10-HP directly coupled with onset of related chemical expansion. The chemical expansion progressively increased with temperature reaching values of 0.75% at 800 °C and 1.28% at 1000 °C. As a consequence, taking into account the elastic modulus of Ce_0.9_Gd_0.1_O_1.95_ of around 180–200 GPa, inhomogeneous re-oxidation can cause very large internal stresses [16,43,44]. For instance, with δ = 0.2 and a related chemical expansion of 2% [15] the arising stress could reach 200 × 0.02 = 4 GPa (4000 MPa). The non-linear dependence of lattice parameter on temperature reflects the development of non-stoichiometry index δ, if linear dependence between them following Bishop et al. is assumed [15].

It must be emphasized, that re-oxidation starts from the sample surface generating tensile stresses in this area. When re-oxidation takes place at room temperature, rapid distribution of oxygen in the lattice is blocked by the reduced diffusion rate of oxygen at room temperature. Therefore, near the sample’s surface a large gradient of oxygen concentration is expected which causes high internal stresses within a few atomic layers. If exceeding a critical value, tensile stresses promote crack formation on the surface, leading to disintegration of the entire sample when propagating into the bulk.

### 3.4. Pressure-Assisted FAST/SPS

**Figure 6:**
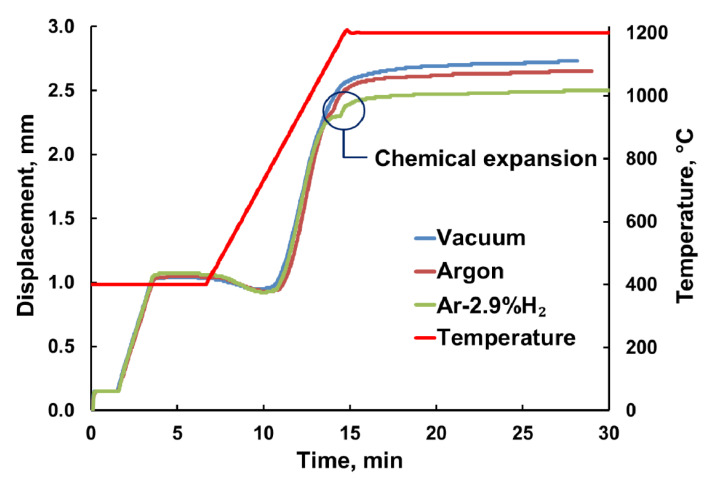
Measured displacement during pressure-assisted FAST/SPS of GDC10-HP powder in different atmospheres.

The effect of atmosphere (vacuum, argon or Ar-2.9%H_2_) during pressure-assisted FAST/SPS sintered was less evident than during sintering in dilatometer (Section 3.1). Densification started at a temperature of around 750 °C in all atmospheres (Figure 6). Also, densification intensity was nearly the same until a temperature of around 1050 °C. Then, in the range of 1050–1150 °C in displacement curves for argon and Ar-2.9%H_2_ a kind of step appeared. During sintering in FAST/SPS device such step is usually an indicator of phase transformation. Displacement measurement with an accuracy of 0.01 mm ensured that this step is not just an instrument error. This effect was the mostly pronounced in the case of sintering in Ar-2.9%H_2_. Contrarily, during sintering in vacuum displacement curve was smooth (Figure 6). We attribute this effect to partial reduction of GDC10-HP with related chemical expansion. The kink in the displacement curve influenced the final density of the related samples. When sintering in vacuum, the highest relative density of 0.963 was observed. Relative density decreased to 0.915 and 0.906 for sintering in argon and Ar-2.9%H_2_, respectively. Apparently, there is a certain relationship between the sintering atmosphere and the final density when keeping all other FAST/SPS parameters constant. However, in this case we did not observe any remarkable influence of sintering atmosphere on resulting grain size.

**Figure 7:**
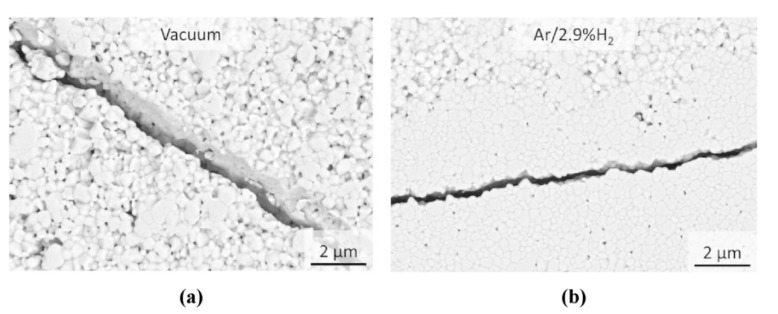
Microstructure of GDC10-HP samples after pressure-assisted FAST/SPS (**a**) in vacuum and (**b**) in Ar-2.9%H_2_.

**Figure 8:**
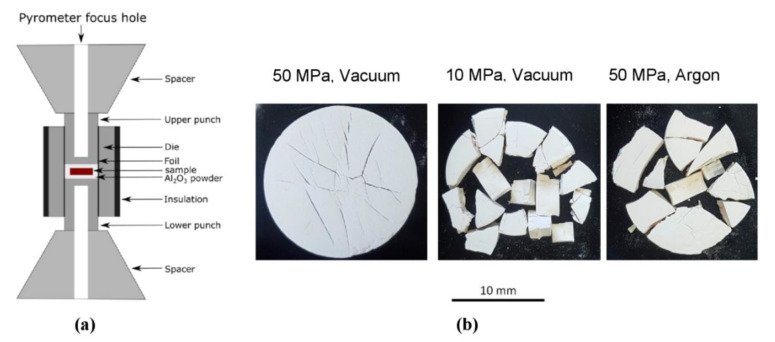
(**a**) Setup and (**b**) broken pellets after sintering of cold-pressed GDC10-HP sample in alumina bed with varying pressure and atmosphere.

Independent on the FAST/SPS atmosphere, all samples were fractured in several pieces. Figure 2d shows a representative sample. When using the standard experimental FAST/SPS setup, re-oxidation at 800 °C did not bring any advantage. Seemingly, feeding air into the FAST/SPS did not change significantly the local environment in the vicinity of the sample. Microstructure of fractured samples is shown in Figure 7. The kind of crack propagation hints towards a preferentially intergranular fracture. The pale yellow color of the starting powder turned to dark grey for all sintered samples (Figure 2d, left). This change in color indicates the partial reduction of GDC10-HP powder during FAST/SPS sintering. As mentioned above, similar result was reported by the research groups, which used micrometer sized CeO_2_ powder [39,40]. The authors stated that fragmentation can be caused by partial reduction of CeO_2_ in contact with graphite and discussed related chemical expansion and abrupt contraction after exposition to air as crucial effects. Despite the fact that this hypothesis is sound, other reasons for disintegration of samples must be taken into consideration as well. Some of them were investigated by additional experiments. In these experiments, we modified the setup to overcome fragmentation, but all attempts failed. In detail, we tested a split die to balance the springback during ejection. Secondly, we significantly reduced the heating and cooling rate to 5 °C/min to exclude cracking by thermal shock due to rapid cooling. Third, we completely removed the load during cooling for reliably avoiding clamping of the sample. Finally, we omitted direct contact of GDC10 to graphite die and graphite foil. For this purpose, a pre-compacted GDC10 sample was completely encapsulated in alumina bed during the FAST/SPS cycle (Figure 8a) following an approach proposed by Prasad as a helpful measure to decrease chemical reduction of CeO_2_ when sintering in graphite tools [41]. However, in our case, even with alumina bed, the sample failed (Figure 8b). We assume that mechanical clamping of GDC10 samples could be the main reason of failure in this case.

### 3.5. Pressureless FAST/SPS

Pressureless FAST/SPS setup (Figure 2c) enabled non-constrained sintering and better atmosphere control in the vicinity of the sample. However, even in this case, during sintering in vacuum, Ce_0.9_Gd_0.1_O_1.95_ is expected to lose a certain amount of oxygen according to reaction (6).

2CeGdO_1.95_ = 2CeGdO_1.95-δ_ + δO_2_ (6)

The value of δ can be evaluated by Equation (7) as reported by Buflin et al. [45]

(7) $$\frac{\delta }{0.35-\delta }=8700\cdot p{\left(O_{2}\right)}^{-0.217}\cdot exp\left(\frac{-195.6\;kJ\cdot mol^{-1}}{RT}\right)$$

Here *p(O_2_)* is the oxygen partial pressure in bar, *R* is the gas constant, and *T* is the absolute temperature. In our experiments, the residual pressure was 0.6 mbar correlating to a *p(O_2_)* of 0.1257 mbar (=0.2095 × 0.6 mbar), and T was 1673 °K. Based on these data, the δ value was calculated to 0.016, which is not a large number. However, we assume that the real δ was higher, leading to a more pronounced non-stoichiometry. As the main reason of this deviation we assume that residual oxygen in the FAST/SPS chamber reacted with the graphite tool resulting in the formation CO, which acts as reducing agent.

**Figure 9:**
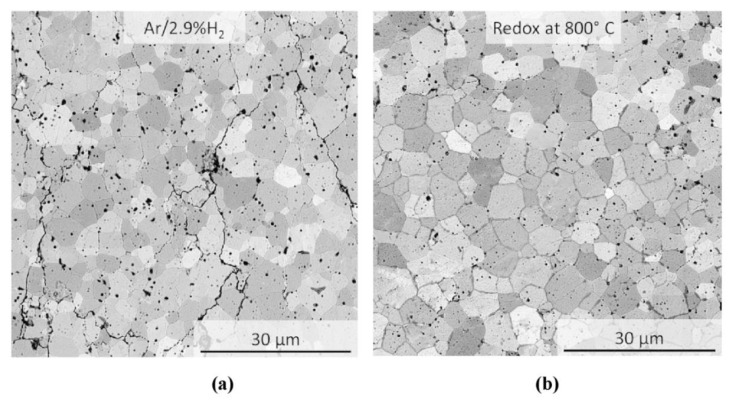
Micrographs of pressureless sintered samples: (**a**) cracks formation after sintering entirely in Ar-2.9%H_2_, (**b**) nearly crack-free structure after sintering in Ar-2.9%H_2_ with subsequent re-oxidation.

**Figure 10:**
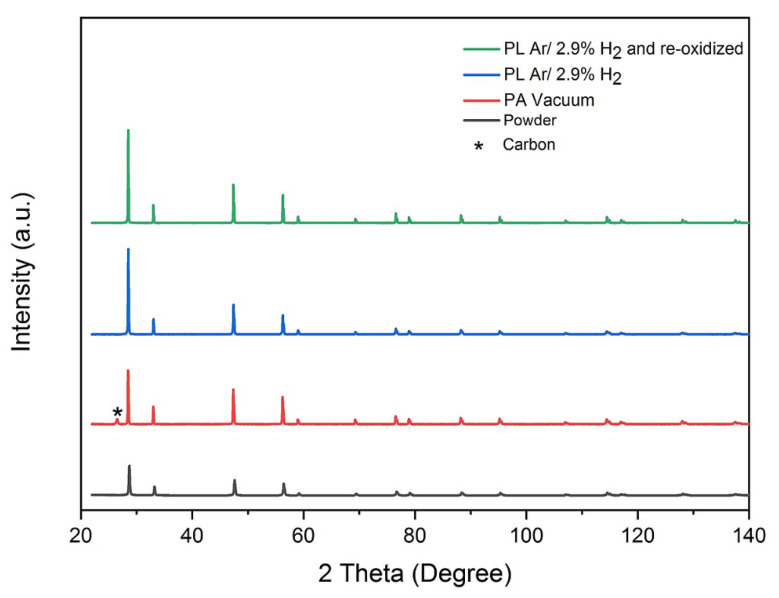
XRD patterns for GDC10-HP samples after FAST/SPS sintering in different atmospheres.

Even if the exact δ value was unknown, the samples sintered in vacuum were mechanically stable. Nevertheless, SEM analysis with backscattered electron detector revealed a large number of cracks in the microstructure (not shown here). Similar result was obtained, when pressureless FAST/SPS was done in Ar-2.9% H_2_ (Figure 9a). In contrast, in this experimental setup re-oxidation at 800 °C was found to be an effective measure to drastically reduce the micro-crack formation (Figure 9b). Nevertheless, the color of sintered samples was dark yellow, indicating still a certain change in stoichiometry (Figure 2d, right). XRD analysis revealed that all sintered samples maintained—with respect to the XRD detection limit—the initial fluorite structure (Figure 10). Other related results are summarized in Table 3. It can be concluded that preventing direct contact to graphite, omitting sample loading and re-oxidation at elevated temperature are required measures to reliably avoid cracking and fragmentation when sintering micrometer sized GDC10-HP powder by FAST/SPS technique.

## 4. Discussion

FAST/SPS is an attractive method for sintering ceramics due to the potential of decreasing sintering temperature and dwell time. Furthermore, it might diminish or avoid decomposition, partial reduction or interfacial reactions of sintered materials. The latter might occur if the ceramic is sintered in direct contact with other functional materials as is, e.g., the case in electrochemical devices. In the present work, we show on the example of GDC that there is a potential to successfully applying FAST/SPS even for sintering of oxide ceramics, which are prone to partial reduction and chemical expansion under FAST/SPS conditions. To take full advantage of this method, further systematic optimization of processing parameters is necessarily needed. Nevertheless, our work clearly shows the critical influencing factors, which have to be taken into careful consideration for establishing and scaling up this method, e.g., for manufacturing of free-standing GDC electrolytes. In the following section, these factors are discussed in detail.

### 4.1. Reduction of Ce_0.9_Gd_0.1_O_1.95_ by Graphite

It is quite evident to assume that Ce_0.9_Gd_0.1_O_1.95_ powder interacts with surrounding graphite tool because carbon is a well-known reducing agent. The reaction between Ce_0.9_Gd_0.1_O_1.95_ and carbon can be formulated as follows:

Ce_0.9_Gd_0.1_O_1.95_ + δC = Ce_0.9_Gd_0.1_O_1.95-δ_ + δCO (8)

**Figure 11:**
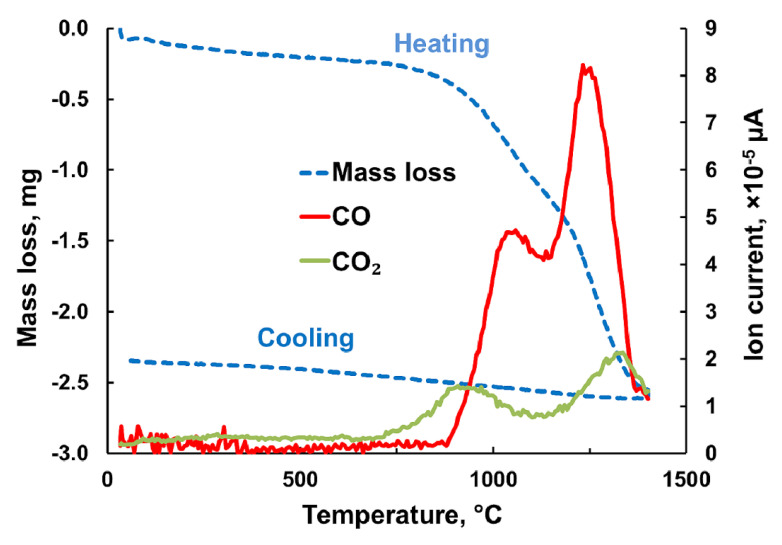
Mass loss and CO/CO_2_ release when heating and cooling a GDC10-HP and carbon black powder mixture in argon.

However, this or similar reaction between pure CeO_2_ and C are hardly reported in the literature. Soller et al. described reaction of CeO_2_ with carbon soot in Ar atmosphere [46]. The authors observed 50% transformations from CeO_2_ to Ce_2_O_3_ already at 500 °C. Brauer reported the reaction between CeO_2_ and C at a temperature of 1000 °C and above as a method for synthesis of Ce_2_O_3_. The author noticed the release of CO and CO_2_ gases during this reaction [47]. In particular, this means that—according to reaction (4)—released CO molecules further accelerate Ce_0.9_Gd_0.1_O_1.95_ reduction. Due to lacking literature data, we preformed own experiments for studying the reaction between GDC10-HP starting powder and carbon. Experimental details regarding thermal gravimetric and spectral analysis were described in Section 2.3. Figure 11 shows the obtained results. Already at a temperature of 600 °C, excessive mass loss by releasing mainly CO and to a lesser extent CO_2_ started. There are two peaks, which are related to desorption of CO species, one at a temperature of around 1035 °C, the other one at 1240 °C. This result clearly evidenced the reaction between GDC10-HP powder and carbon in the temperature range of interest for sintering GDC by FAST/SPS. For better underfunding, this reaction must be studied in more detail. It should be emphasized, that reduction of Ce_0.9_Gd_0.1_O_1.95_ leads to formation of oxygen vacancies according to reaction (2) coupled with increase of sintering kinetics as shown before.

### 4.2. Electrochemical Reduction of Ce_0.9_Gd_0.1_O_1.95_

Electrochemical reactions in an electric field with constant polarity can result in additional reduction of Ce_0.9_Gd_0.1_O_1.95_. This phenomenon was mainly studied in Flash Sintering modus, where the electric field strength is at least one order of magnitude higher than in FAST/SPS [48]. However, some preliminary investigations on electrochemical reduction of CeO_2_ in FAST/SPS modus are also available in literature [41]. In general, the electrochemical reduction of Ce_0.9_Gd_0.1_O_1.95_ can be described by two half reactions. On the negative electrode (the cathode, where the material is reduced), a part of cerium ions adopts electrons causing a valence change from Ce^+4^ to Ce^+3^ in the fluorite lattice. The valence change is accompanied by release of O^2-^ ions and formation of oxygen vacancies. This process is described by the half reaction (9).

Ce_0.9_Gd_0.1_O_1.95_ + 2δe^−1^ = Ce_0.9_Gd_0.1_O_1.95−δ_ + δO^2−^ (9)

The oxygen ions migrate under electrostatic force towards the positive electrode (the anode, where the material is re-oxidized). Here, electrons are released with formation of oxygen molecules in accordance with half reaction (10).

δO^2−^ − 2δe^−1^ = 1/2δO_2_ (10)

In sum, the entire redox reaction is

Ce_0.9_Gd_0.1_O_1.95_ = Ce_0.9_Gd_0.1_O_1.95-δ_ + δ/2O_2_ (11)

Reduction of GDC increases its electronic conductivity based on a polaron hopping mechanism. With ongoing dwell time, the reduced area spreads through the sample with remarkable change in color, which is known in the literature as “blackening effect” [17]. As an undesirable side effect, a quite inhomogeneous stoichiometry of the sample might result. Prasad observed such heterogeneous stoichiometry by a gradient in coloration in a CeO_2_ sample sintered by FAST/SPS [41]. He also noticed formation of certain porosity in the sample volume near to the negative electrode (cathode). It is assumed that strongly pronounced generation of vacancies on the negative electrode is the main reason for appearing porosity, as described above. In spite of having first results, electrochemical reduction of pure and doped ceria under FAST/SPS conditions still need more detailed investigations.

### 4.3. Prospects for Application of FAST/SPS for Sintering of Ce_0.9_Gd_0.1_O_1.95_

In this work we demonstrated that crack-free FAST/SPS sintering of dense Ce_0.9_Gd_0.1_O_1.95_ pellets in a conventional setup is a challenging task and potentially requires adapted tool design. The main reason for that is the reduction of Ce_0.9_Gd_0.1_O_1.95_ due to direct contact with graphite tool or graphite foil and due to electrochemical reactions on the electrodes. At a direct contact to graphite, we assume that carbon diffuses mainly along grain boundaries, causing a more pronounced stoichiometry change near the grain boundaries. This effect might weaken the grain boundaries as compared to the bulk. The reduction and the change in stoichiometry are accompanied with chemical expansion. These processes become significantly intensified with increase of temperature. Similar behavior was observed during thermal treatment of Ce_0.8_Gd_0.2_O_1.9_ in hydrogen at high temperature [12]. When reduced Ce_0.9_Gd_0.1_O_1.95_ pellet is exposed to air, sudden re-oxidation occurs with rapid and inhomogeneous change in stoichiometry. This leads to gradients in chemical contraction and to related stress generation. If stress exceeds a critical value, the cracks emerge, resulting in fragmentation of pellets primarily along weak grain boundaries. Thus, the main strategy for crack-free sintering of doped ceria by FAST/SPS technique is diminishing both chemical and electrochemical reduction. This can be achieved by decrease in sintering temperature and sintering time. A well-known approach for lowering of sintering temperature is the use of nanometeric powders with exceptional high sintering activity. For ceria-based materials, the required particle size below 20 nm is reported in the literature [33,35,36,37,38]. If powder with a larger particle size (approximately up to 100 nm) is used, the application of a pressure is necessary, which by far exceeds typical pressures in standard FAST/SPS. In this context, pressures in the range of 500–600 MPa are recommended [34]. The more radical measure is to prevent the contact between sintered material and graphite component of tool. An example is the sintering in alumina (or other ceramic) powder bed as proposed by Prasad [41]. This approach can be used for sintering of micrometer sized powders, which require an enhanced sintering temperature in a range of 1200–1400 °C. However, our experience has shown that this method of sintering does not always work. Excluding a clamping effect in FAST/SPS die is additionally required to omit or to minimize the crack formation. In this paper we proposed the use of pressureless sintering of cold-pressed pellets in a specially designed FAST/SPS tool. Such a scheme of sintering was used before by some other researchers. The detailed review on this sintering technique was recently published by Yamanoglu [49]. The main application of pressureless sintering deals with the synthesis of porous materials. Besides, high heating rate resulting in fine grained structure is an additional advantage of this sintering method. Furthermore, we believe that there is a potential for application of pressureless FAST/SPS to sintering of free-standing thin ceramic membranes produced, e.g., by tape casting. If applying this technology for Ce_0.9_Gd_0.1_O_1.95_ or other similar materials, which are prone to oxygen release and chemical expansion, the most important need is the reliable control of atmosphere inside FAST/SPS setup. In a standard FAST/SPS tool such a control is practically impossible, because the sample is tightly encapsulated in the die. The use of moderate vacuum typical for FAST/SPS cannot secure the integrity of GDC sample even in open to FAST/SPS chamber setup. In our opinion, this could be due to reaction of residual oxygen with graphite at temperature above 600 °C, leading to the formation of CO. Release of CO accelerates reduction of Ce_0.9_Gd_0.1_O_1.95_ and chemical expansion at sintering temperature, finally resulting in fragmentation of the sample. A way to overcome crack formation is controlled re-oxidation at elevated temperatures, e.g., at 800 °C. At such temperature Ce_0.9_Gd_0.1_O_1.95_ still behaves like a viscous solid [50] with possibility to release internal stresses arising during re-oxidation. Another option for crack-free sintering of Ce_0.9_Gd_0.1_O_1.95_ could be the application of a carbon-free tool, e.g., made of TZM, cemented carbide or conductive ceramics. The graphite foil also must be replaced by a foil non-interacting with Ce_0.9_Gd_0.1_O_1.95_. This approach is the matter of our ongoing research. In our future work, we plan to transfer the developed method of FAST/SPS sintering with controlled atmosphere to other electrochemically active oxides for oxygen transport membranes like Ba_x_Sr_1-x_Co_y_Fe_1-y_O_x-d_ (BSCF) or La_x_Sr_1-x_Co_y_Fe_1-y_O_x-d_ (LSCF), which showed cracking behavior similar to GDC [51]. Another goal is to sinter free-standing Li_7_La_3_Zr_2_O_12_ (LLZ) and Li_1.5_Al_0.5_Ti_1.5_(PO_4_)_3_ (LATP) electrolytes for all-solid-state batteries starting from tape casted components and scaling up the technology by adapting the proposed tool design. At the end of the paper the newly reported application of cold sintering technique for consolidation of GDC powder should be mentioned [52]. This technique requires sintering temperatures far below the onset temperature for GDC reduction. Thus, cold sintering can be combined with FAST/SPS technique in one technological step as reported earlier for other materials [53].

## 5. Conclusions

In the present work, we performed a detailed literature study, experimental investigation and discussion of special features related to FAST/SPS sintering of Ce_0.9_Gd_0.1_O_1.95_ (GDC) powder. GDC was chosen as representative for oxide materials, which are prone to oxygen release (i.e., reduction), coupled with significant chemical expansion. The main findings of this work enabled the following main conclusions.

Both literature data and our experiments reveal that application of a reducing atmosphere during free sintering significantly enhances the densification kinetics of GDC powder and grain growth if compared to sintering in air. In particular, this was evidenced by lowering the onset temperature of densification while keeping all other sintering parameters unchanged. A similar, but less pronounced, effect was observed in our experiments in the case of free sintering of GDC in argon. However, the use of atmospheres with different reducing potential (e.g., Ar-2.9%H_2_, argon and middle vacuum) in the chamber of the FAST/SPS device did not result in any significant effect on densification kinetics and grain growth under FAST/SPS conditions. This means that sintering of GDC via FAST/SPS is mainly determined by local, difficult to control environment in the cavity of the graphite tool, which is dominated by the presence of carbon including its direct contact with the sample.

Heating or sintering in a reducing atmosphere changes the stoichiometry of GDC powder with related chemical expansion. In our high-temperature XRD experiments we found that this process started at about 500 °C during heating in Ar-2.9%H_2_. For the GDC10-HP powder used in this study, a chemical expansion of more than 1.2% at 1000 °C was observed. During FAST-SPS sintering, we identified chemical expansion of GDC as a kink in densification curves. This observation evidenced the change of stoichiometry, i.e., reduction of GDC during FAST-SPS processing. The interaction between GDC and carbon was additionally confirmed by a thermal gravimetric study.

When exposing GDC in the reduced state to air, rapid re-oxidation and contraction of material arises. This effect leads to large internal stresses causing formation of intergranular cracks and fragmentation of sintered solids. This well-known in free sintering effect was also observed in our FAST/SPS experiments with micrometer sized GDC10-HP powder. To suppress reduction of GDC and to omit related cracking during FAST/SPS, we conclude that avoiding direct contact of sintered powder with graphite foil in a special designed tool is the key to reliable sintering oxides prone to chemical expansion. In addition, the new tool excludes clamping of samples between punches (electrodes) and provides control of atmosphere in vicinity of the sintered sample. This possibility was used to re-oxidize the GDC sample by synthetic air at 800 °C. With all of these measures, we were able to fully avoid fragmentation of GDC samples and to achieve nearly crack-free sintering. We believe that this concept enables the upscaling of FAST/SPS technology toward sintering of larger GDC components.

In future, the developed FAST/SPS approach will be applied for sintering other active oxides prone to chemical expansion, such as BSCF and LSCF for oxygen transport membranes or LLZ and LATP as electrolyte for all-solid-state-batteries (ASSB).

## Figures and Tables

**Table 1:** Literature survey of Field-Assisted Sintering (FAST/SPS) of undoped and doped ceria. In these works, the authors did not report any crack formation or fragmentation of samples.

Material	Manufacturer	Particle Size	Temperature	Pressure	Holding	Density	Reference
		nm	°C	MPa	Min.	%	
CeO_2_	Not reported	<10	625	600	5	>98	[33]
Ce_0.7_Sm_0.3_O_1.85_	Not reported	<10	750	610	5	>98	[33]
Ce_0.9_Dy_0.1_ O_1.95_	In-house	73	1050	500	5	97	[34]
Ce_0.8_Dy_0.2_O_1.9_	In-house	20	1200	60	15	85	[30]
Ce_0.8_Sm_0.2_O_1.9_	In-house	7–11	1000	150	10	95	[35]
Ce_0.8_Gd_0.2_O_1.9_	In-house	5–7	1050	150	5	>98.7	[36]
Ce_0.8_Gd_0.2_O_1.9_	In-house	<20	900	90	5	96.2	[25]
Ce_0.9_Gd_0.1_O_1.95_	In-house	12	980	70	5	>96	[38]

**Table 2:** Literature survey of FAST/SPS of undoped ceria. In these works, the authors report crack formation or disintegration of samples.

Material	Manufacturer	Particle Size	Temperature	Pressure	Holding	Density	Reference
		µm	°C	MPa	Min.	%	
CeO_2_	Sigma-Aldrich	<5	1500	80	3	91	[39]
CeO_2_	ACROS Organics	20	1400	50	5	91	[40]

**Table 3:** Parameters and results of pressure-assisted and pressureless FAST/SPS sintering of GDC10-HP powder.

Sample ID ^*^	Temperature, °C	Dwell, min	Atmosphere	Rel. Density	Integrity
PAS-1	1200	2	Vacuum	0.95	Fracture
PAS-2	1200	2	Ar-2.9%H_2_	0.94	Fracture
PAS-3	1200	2	Ar-2.9%H_2_ + re-oxidation	0.94	Fracture
PLS-1	1400	15	Vacuum	0.92	Micro-cracks
PLS-2	1400	15	Ar-2.9%H_2_	0.91	Micro-cracks
PLS-3	1400	15	Ar-2.9%H_2_ + re-oxidation	0.93	Nearly crack-free

* PAS denotes pressure-assisted sintering. PLS designates pressureless sintering.

## References

[1] Hennings U., Reimert R. (2007). Investigation of the structure and the redox behavior of gadolinium doped ceria to select a suitable composition for use as catalyst support in the steam reforming of natural gas. Appl. Catal. A.

[2] Brandon N.P., Corcoran D., Cummins D., Duckett A., El-Khoury K., Haigh D., Leah R., Lewis G., Maynard N., McColm T. (2004). Development of metal supported solid oxide fuel cells for operation at 500–600 °C. J. Mater. Eng. Perform..

[3] Lee J., Park J., Shul Y. (2014). Tailoring gadolinium-doped ceria-based solid oxide fuel cells to achieve 2 W∙cm^−2^ at 550  °C. Nat. Commun..

[4] Ramasamy M., Baumann S., Palisaitis J., Schulze-Küppers F., Balaguer M., Kim D., Meulenberg W.A., Mayer J., Bhave R., Guillon O. (2016). Influence of microstructure and surface activation of dual-phase membrane Ce_0.8_Gd_0.2_O_2-δ_–FeCo_2_O_4_ on oxygen permeation. J. Am. Ceram. Soc..

[5] Rojek-Wöckner V.A., Opitz A.K., Brandner M., Mathé J., Bram M. (2016). A novel Ni/ceria based anode for metal-supported solid oxide fuel cells. J. Power Sources.

[6] Bischof C., Nenning A., Malleier A., Martetschläger L., Gladbach A., Schafbauer W., Opitz A.K., Bram M. (2019). Microstructure optimization of nickel/gadolinium-doped ceria anodes as key to significantly increasing power density of metal-supported solid oxide fuel cells. Int. J. Hydrogen Energy.

[7] Udomsilp D., Thaler F., Menzler N.H., Bischof C., de Haart L.G.J., Opitz A.K., Guillon O., Bram M. (2019). Dual-phase cathodes for metal-supported solid oxide fuel cells. – Processing, performance, durability. J. Electrochem. Soc..

[8] Steele B.C.H. (2000). Appraisal of Ce_1-y_Gd_y_O_2-y/2_ electrolytes for IT-SOFC operation at 500 °C. Solid State Ion..

[9] Fierro J.L.G., Soria J., Sanz J., Rojo J.M. (1987). Induced changes in ceria by thermal treatments under vacuum or hydrogen. J. Solid State Chem..

[10] Otsuka K., Hatano M., Morikawa A. (1983). Hydrogen from water by reduced cerium oxide. J. Catal..

[11] Kim K.J., Choi G.M. (2015). Phase stability and oxygen non-stoichiometry of Gd-doped ceria during sintering in reducing atmosphere. J. Electroceram..

[12] Badwal S.P.S., Fini D., Ciacchi F.T., Munnings C., Kimpton J.A., Drennan J. (2013). Structural and microstructural stability of ceria–gadolinia electrolyte exposed to reducing environments of high temperature fuel cells. J. Mater. Chem. A.

[13] Hong S.J., Virkar A.V. (1995). Lattice parameters and densities of rare-earth oxide doped ceria electrolytes. J. Am. Ceram. Soc..

[14] Brauer G., Gingerich K.A. (1960). Über die Oxyde des Cers—V. Hochtemperatur-Röntgenuntersuchungen an ceroxyden. J. Inorg. Nucl. Chem..

[15] Bishop S.R., Duncan K.L., Wachsman E.D. (2009). Defect equilibria and chemical expansion in non-stoichiometric undoped and gadolinium-doped cerium oxide. Electrochim. Acta.

[16] Krishnamurthy R., Sheldon B.W. (2004). Stresses due to oxygen potential gradients in non-stoichiometric oxides. Acta Mater..

[17] Janek J., Korte C. (1999). Electrochemical blackening of yttria-stabilized zirconia—Morphological instability of the moving reaction front. Solid State Ionics.

[18] Bevan D.J.M. (1955). Ordered intermediate phases in the system CeO_2_-Ce_2_O_3_. J. Inorg. Nucl. Chem..

[19] Neuhaus K., Dolle R., Wiemhöfer H.D. (2020). The effect of transition metal oxide addition on the conductivity of commercially available Gd-doped ceria. J. Electrochem. Soc..

[20] Ni D.W., De Florio D.Z., Marani D., Kaiser A., Tinti V.B., Esposito V. (2015). Effect of chemical redox on Gd-doped ceria mass diffusion. J. Mater. Chem. A.

[21] Ni D.W., Glasscock J.A., Pons A., Zhang W., Prasad A., Sanna S., Pryds N., Esposito V. (2014). Densification of highly defective ceria by high temperatures controlled reoxidation. J. Electrochem. Soc..

[22] He Z., Yuan H., Glasscock J.A., Chatzichristodoulou C., Phair J.W., Kaiser A., Ramousse S. (2010). Densification and grain growth during early-stage sintering of Ce_0.9_Gd_0.1_O_1.95-δ_ in a reducing atmosphere. Acta Mater..

[23] Esposito V., Ni D.W., He Z., Zhang W., Prasad A.S., Glasscock J.A., Chatzichristodoulou C., Ramousse S., Kaiser A. (2013). Enhanced mass diffusion phenomena in highly defective doped ceria. Acta Mater..

[24] German R. (2014). Sintering: From Empirical Observations to Scientific Principles.

[25] Shimonosono T., Sakka Y., Hirata Y. (2009). Fast low-temperature consolidation of bulk nanometric ceramic materials. Trans. Mater. Res. Soc. Jpn..

[26] Guillon O., Gonzalez-Julian J., Dargatz B., Kessel T., Schierning G., Räthel J., Herrmann M. (2014). Field-assisted sintering technology/spark plasma sintering: Mechanisms, materials, and technology developments. Adv. Eng. Mater..

[27] Tokita M. (2019). Spark Plasma Sintering: Method, systems, applications and industrialization. Powder Metall. Rev..

[28] Suárez M., Fernández A., Menéndez J.L., Torrecillas R., Kessel H.U., Hennicke J., Kirchner R., Kessel T., Ertug B. (2013). Challenges and opportunities for Spark Plasma Sintering: A key technology for a new generation of materials. Sintering Applications.

[29] Vanmeensel K., Laptev A., Sheng H., Tkachenko I., Van der Biest O., Vleugels J. (2013). Experimental study and simulation of plastic deformation of zirconia-based ceramics in a pulsed electric current apparatus. Acta Mat..

[30] Laptev A.M., Zheng H., Bram M., Finsterbusch M., Guillon O. (2019). High-pressure field assisted sintering of half-cell for all-solid-state battery. Mater. Lett..

[31] Groeneveld D., Groß H., Hansen A.L., Dankwort T., Hansen J., Wöllenstein J., Bensch W., Kienle L., König J. (2019). High-pressure sintering of rhombohedral Cr_2_S_3_ using titanium-zirconium-molybdenum tools. Adv. Eng. Mater..

[32] Wang K.S., Tan J.F., Hu P., Yu Z.T., Yang F., Hu B.L., Song R., He H.C., Volinsky A.A. (2015). La_2_O_3_ effects on TZM alloy recovery, recrystallization and mechanical properties. Mat. Sci. Eng. A..

[33] Anselmi-Tamburini U., Garay J.E., Munir Z.A. (2006). Fast low-temperature consolidation of bulk nanometric ceramic materials. Scripta Mater..

[34] Choi K., Reavis R.E., Osterberg D.D., Jaques B.J., Butt D.P., Mariani R.D., Burkes D.E., Munir Z.A. (2012). Effect of dysprosia additive on the consolidation of CeO_2_ by Spark Plasma Sintering. J. Am. Ceram. Soc..

[35] Mori T., Kobayashi T., Wang Y. (2005). Synthesis and characterization of nano-hetero-structured Dy-doped CeO_2_ solid electrolytes using a combination of spark plasma sintering and conventional sintering. J. Am. Ceram. Soc..

[36] Solodkyi I., Borodianska H., Sakka Y., Vasylkiv O. (2012). Effect of grain size on the electrical properties of samaria-doped ceria solid electrolyte. J. Nanosci. Nanotechnol..

[37] Vasylkiv O., Borodianska H., Sakka Y. (2008). Nanoreactor engineering and SPS densification of multimetal oxide ceramic nanopowders. J. Eur. Ceram. Soc..

[38] Kabir A., Santucci S., Nong N.V., Varenik M., Lubomirsky I., Nigon R., Muralt P., Esposito V. (2019). Effect of oxygen defects blocking barriers on gadolinium doped ceria (GDC) electro-chemo-mechanical properties. Acta Mater..

[39] Watkinson E.J., Ambrosi R.M., Kramer D.P., Williams H.R., Reece M.J., Chen K., Sarsfield M.J., Barklay C.D., Fenwick H., Weston D.P. (2017). Sintering trials of analogues of americium oxides for radioisotope power systems. J. Nucl. Mater..

[40] Prasad A., Malakkal L., Bichler L., Szpunar J., Bansal N.P., Castro R.H.R., Jenkins M., Bandyopadhyay A., Bose S., Bhalla A., Singh J.P., Mahmoud M.M., Pickrell G., Johnson S. (2018). Challenges in spark plasma sintering of cerium (IV) oxide. Processing of Properties, and Design of Advanced Ceramics and Composites II: Ceramic Transactions.

[41] Prasad A.R. (2017). Spark Plasma Sintering of Cerium Dioxide and its Composites. Master’s Thesis.

[42] Laptev A.M., Bram M., Vanmeensel K., Gonzalez-Julian J., Guillon O. (2018). Enhancing efficiency of field assisted sintering by advanced thermal insulation. J. Mater. Process. Tech..

[43] Yavo N., Noiman D., Wachtel E., Kim S., Feldman Y., Lubomirsky L., Yeheskel O. (2016). Elastic moduli of pure and gadolinium doped ceria revisited: Sound velocity measurements. Scripta Mat..

[44] Amezawa K., Kushi T., Sato K., Unemoto A., Hashimoto S., Kawada T. (2011). Elastic moduli of Ce_0.9_Gd_0.1_O_2−δ_ at high temperatures under controlled atmospheres. Solid State Ionics.

[45] Bulfin B., Lowe A.J., Keogh K.A., Murphy B.E., Lübben O., Krasnikov S.A., Shvets I.V. (2013). Analytical model of CeO_2_ oxidation and reduction. J. Phys. Chem. C..

[46] Soler L., Casanovas A., Escudero C., Pérez-Dieste V., Aneggi E., Alessandro Trovarelli A., Llorca J. (2016). Ambient pressure photoemission spectroscopy reveals the mechanism of carbon soot oxidation in ceria-based catalysts. ChemCatChem.

[47] Brauer G., Brauer G. (1978). Selten-Erd-Metalle. Handbuch der Präparativen Anorganischen Chemie.

[48] Mishra T.P., Neto R.R.I., Speranza G., Quaranta A., Sgalvo V.M., Raj R., Guillon O., Bram M., Biesuz M. (2020). Electronic conductivity in Gadolinium doped ceria under DC bias as a trigger for flash sintering. Scripta Mat..

[49] Yamanoglu R. (2019). Pressureless spark plasma sintering: A perspective from conventional sintering to accelerated sintering without pressure. Powder Metall. Met. Ceram..

[50] Chang J., Guillon O., Rödel J., Kang S.K.L. (2007). Uniaxial viscosity of gadolinium-doped ceria determined by discontinuous sinter forging. J. Eur. Ceram. Soc..

[51] Laptev A., Bram M., Zivcec M., Baumann S., Jarligo M.O., Sebold D., Pfaff E., Broeckmann C. (2013). Manufacturing of metal supported BSCF membranes by spark plasma sintering. In Metal Supported Membranes. Proceedings of the European Powder Metallurgy Congress.

[52] Kabir A., Espineira Cachaza M., Fiordaliso E.M., Ke D., Grasso S., Merle B., Esposito V. Effect of cold sintering process (CSP) on the electro-chemo-mechanical properties of Gd-doped ceria (GDC). J. Eur. Ceram. Soc..

[53] Pereira da Silva J.G., Bram M., Laptev A.M., Gonzalez-Julian J., Ma Q., Tietz F., Guillon O. (2019). Sintering of a sodium-based NASICON electrolyte: A comparative study between cold, field assisted and conventional sintering methods. J. Eur. Ceram. Soc..

